# Cobalt oxide nanoparticles aggravate DNA damage and cell death in eggplant via mitochondrial swelling and NO signaling pathway

**DOI:** 10.1186/s40659-016-0080-9

**Published:** 2016-03-18

**Authors:** Mohammad Faisal, Quaiser Saquib, Abdulrahman A. Alatar, Abdulaziz A. Al-Khedhairy, Mukhtar Ahmed, Sabiha M. Ansari, Hend A. Alwathnani, Sourabh Dwivedi, Javed Musarrat, Shelly Praveen

**Affiliations:** Department of Botany and Microbiology, College of Sciences, King Saud University, P.O Box 2455, Riyadh, 11451 Saudi Arabia; A.R. Al-Jeraisy Chair for DNA Research, Zoology Department, College of Sciences, King Saud University, P.O. Box 2455, Riyadh, 11451 Saudi Arabia; Zoology Department, College of Sciences, King Saud University, P.O Box 2455, Riyadh, 11451 Saudi Arabia; Department of Agricultural Microbiology, Faculty of Agricultural Sciences, AMU, Aligarh, 202002 India; Department of Biosciences and Biotechnology, Baba Ghulam Shah Badshah University, Rajouri, 185131 Jammu and Kashmir India; Division of Plant Pathology, Indian Agricultural Research Institute, New Delhi, 110012 India

**Keywords:** Cobalt oxide nanoparticles, Nanotoxicity, DNA damage, Apoptosis, Oxidative stress

## Abstract

**Background:**

Despite manifold benefits of nanoparticles (NPs), less information on the risks of NPs to human health and environment has been studied. Cobalt oxide nanoparticles (Co_3_O_4_-NPs) have been reported to cause toxicity in several organisms. In this study, we have investigated the role of Co_3_O_4_-NPs in inducing phytotoxicity, cellular DNA damage and apoptosis in eggplant (*Solanum melongena* L. cv. Violetta lunga 2). To the best of our knowledge, this is the first report on Co_3_O_4_-NPs showing phytotoxicity in eggplant.

**Results:**

The data revealed that eggplant seeds treated with Co_3_O_4_-NPs for 2 h at a concentration of 1.0 mg/ml retarded root length by 81.5 % upon 7 days incubation in a moist chamber. Ultrastructural analysis by transmission electron microscopy (TEM) demonstrated the uptake and translocation of Co_3_O_4_-NPs into the cytoplasm. Intracellular presence of Co_3_O_4_-NPs triggered subcellular changes such as degeneration of mitochondrial cristae, abundance of peroxisomes and excessive vacuolization. Flow cytometric analysis of Co_3_O_4_-NPs (1.0 mg/ml) treated root protoplasts revealed 157, 282 and 178 % increase in reactive oxygen species (ROS), membrane potential (Δ*Ψm*) and nitric oxide (NO), respectively. Besides, the esterase activity in treated protoplasts was also found compromised. About 2.4-fold greater level of DNA damage, as compared to untreated control was observed in Comet assay, and 73.2 % of Co_3_O_4_-NPs treated cells appeared apoptotic in flow cytometry based cell cycle analysis.

**Conclusion:**

This study demonstrate the phytotoxic potential of Co_3_O_4_-NPs in terms of reduction in seed germination, root growth, greater level of DNA and mitochondrial damage, oxidative stress and cell death in eggplant. The data generated from this study will provide a strong background to draw attention on Co_3_O_4_-NPs environmental hazards to vegetable crops.

## Background

Over a last decade, nanotechnology has gained an immense research interest due to its applications in public health, medicine, industry and agriculture. The incessant use of nanoparticles (NPs) in a multitude of sectors presents a risk of their release into the environment, which may pose serious threats on ecosystem and adversely affect its living entity [1]. Particularly, plants are at maximum risk due to the concentration build-up of NPs in natural sediments, agricultural soils, and aquatic environments [1, 2]. Recent evidences on the NPs toxicity demonstrated the cellular uptake of Ag-NPs in *Oryza Sativa* and Cu/CuO-NPs in *Lactuca sativa* [3, 4]. *Vicia faba* exposed to multiwalled carbon nano tubes exhibited imbalance of nutrient elements, leaves damage and oxidative stress [5]. The uptake and translocation of TiO_2_-NPs in *Allium cepa* induces heavy ROS generation, sticky, multipolar and laggard chromosomes, including micronucleus formation and DNA damage [6]. These effects of NPs are primarily associated with their increased surface area and reactivity, ROS generation and the tendency to form agglomerates [4]. We have selected Co_3_O_4_-NPs for the current investigation due to its unique physical properties, applications in pigments, catalysis, sensors, electrochemistry, magnetism and energy storage [7]. In addition, the composites of Co_3_O_4_-NPs with multiwalled carbon nanotubes have been proposed for fabricating high-performance electronic devices [8].

Till date, only a solitary report on Co_3_O_4_-NPs demonstrated the reduction of root length in *A. cepa*, without much elaboration on the nature of cellular damage and mechanism of the phytotoxicity [9]. Therefore, in this study, we have investigated the mechanistic aspects of Co_3_O_4_-NPs toxicity in eggplant (*Solanum melongena* L. cv. Violetta lunga 2), an economically important vegetable crop, as a model, using state-of-the-art techniques like transmission electron microscopy (TEM), comet assay and flow cytometry. This will help in understanding as to how the plant responds to NPs exposure and regulates the molecular mechanism of cell death pathways. Since no systematic study has been attempted so far, describing the mechanism of Co_3_O_4_-NPs induced phytotoxicity in eggplant at cellular and molecular levels, we have investigated the effect of Co_3_O_4_-NPs on eggplant cells to assess the (1) phytotoxicity, (2) translocation of Co_3_O_4_-NPs in root cells and subcellular anomalies, (3) intracellular ROS generation and mitochondrial dysfunction (Δ*Ψm*), (4) DNA damage (5) cell cycle alterations, NO generation and esterase activity.

## Results and discussion

### Co_3_O_4_-NPs characterization

**Fig. 1:**
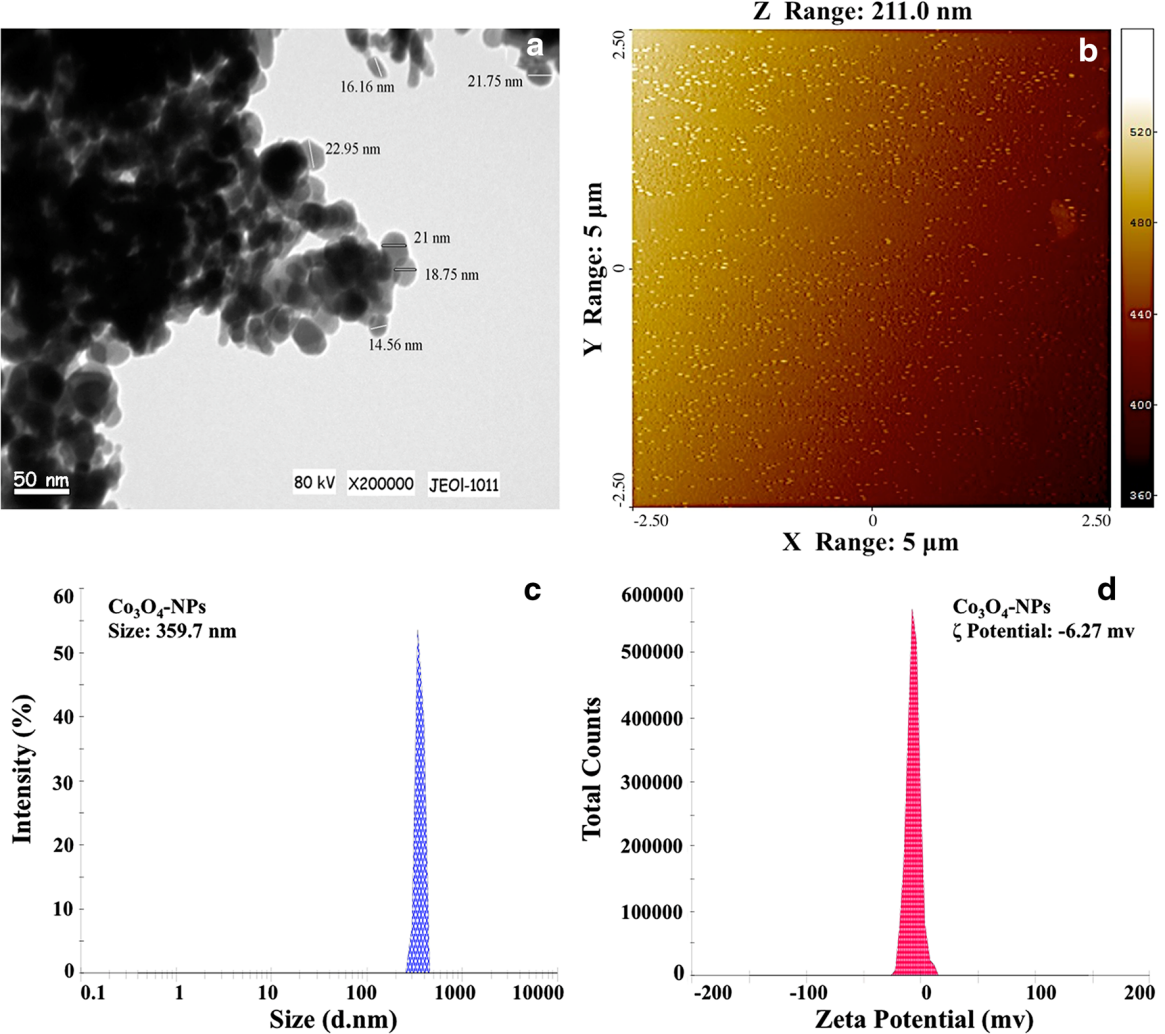
TEM analysis of Co_3_O_4_-NPs (**a**) at ×200,000 magnification. **b** Depicts a topography of Co_3_O_4_-NPs in an AFM perspective. Dynamic light scattering (DLS) and ζ-potential analysis of Co_3_O_4_-NPs suspension in ultrapure water (**c,** **d**)

Size and morphology of Co_3_O_4_-NPs were examined by TEM and AFM analyses. TEM image in Fig. 1 revealed the morphology of Co_3_O_4_-NPs as crystallite spheres with polyhedral structure, and aggregates have been observed. The average particle size of Co_3_O_4_-NPs determined from six different TEM images were 21.3 nm (Fig. 1a). AFM analysis of Co_3_O_4_-NPs exhibited the size of NPs to be 40 nm (Fig. 1b). Since, the sizes of NPs are regarded as important parameters for cellular toxicity; the behavior of Co_3_O_4_-NPs in treatment solutions was examined through DLS, to understand the extent of aggregation and secondary size of NPs. The data revealed large particle aggregates of 359.7 ± 2.1 nm with ζ-potential of −6.2 ± 1.3 mv (Fig. 1c, d). DLS is widely used to determine the size of Brownian NPs in colloidal suspensions in the nano and submicron ranges [10]. The average hydrodynamic particle’s diameter in water indicates particle aggregation. Our characterization data corroborates well with the recent report on agglomeration of NPs in an aqueous environment [3].

### Co_3_O_4_-NPs treatment retarded the root growth of eggplant

**Fig. 2:**
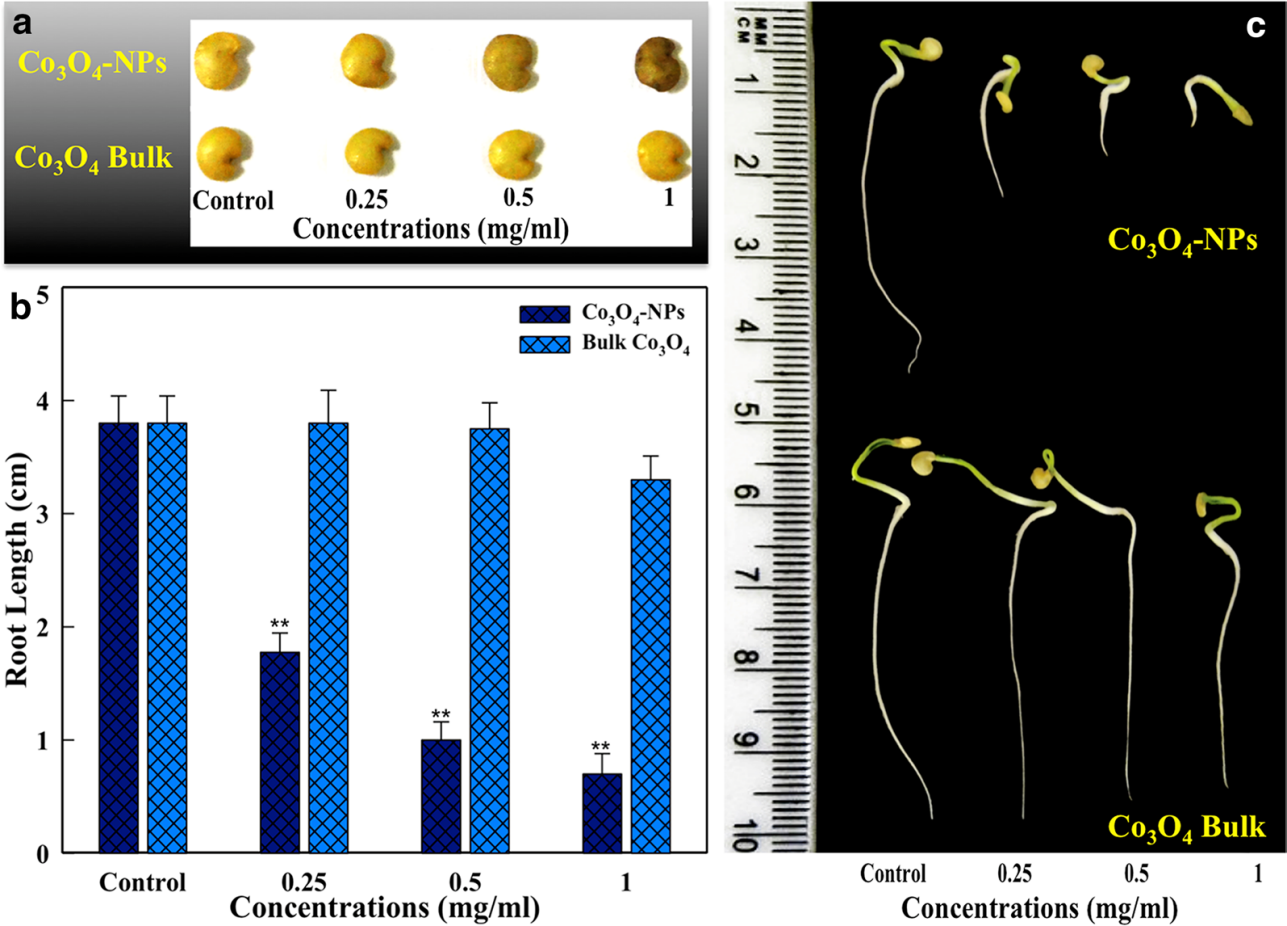
**a** Depicts the adsorption of Co_3_O_4_-NPs and bulk Co_3_O_4_ on eggplant seeds after 2 h of exposure. Concentration dependent repression of average root length (**b**) of eggplant (**p < 0.01 vs. control). Phenotypic changes showing stunting and thickening of eggplant seedling roots after 7 days of exposure with Co_3_O_4_-NPs, while the bulk Co_3_O_4_ groups exhibited normal morphology of seedling roots (**c**)

Co_3_O_4_-NPs treated seeds exhibited a tendency to adsorb on the seed coat in a concentration dependent manner, while bulk Co_3_O_4_ did not display adsorption behavior on the seeds (Fig. 2a). These observations are in line with our previous report exhibiting the adsorption tendency NiO-NPs on the tomato seeds [11]. Such behavior could be due to the physical attachment of particles on a rough seed surface, electrostatic attraction and hydrophobic interactions between seeds and NP agglomerates. It is likely that the adsorption of NPs may facilitate the release of ions locally to enhance phytotoxic effects [11]. Exposure concentrations for Co_3_O_4_-NPs were selected based on the screening of low to high concentrations (0.025–1.0 mg/ml) of Co_3_O_4_-NPs to affect the root length. Since, the primary objective of this study was to assess the toxicity of Co_3_O_4_-NPs. Therefore, the non-significant concentrations (0.025–0.1 mg/ml) in terms of repression of root lengths were excluded from further toxicity experiments. After 7 days of incubation, average root lengths recorded at 0.5 and 1.0 mg/ml of Co_3_O_4_-NPs were 1.0 ± 0.16 and 0.70 ± 0.18 cm (p < 0.01) respectively. The root length in untreated control was 3.80 ± 0.24 cm (Fig. 2b). Under similar experimental conditions, bulk Co_3_O_4_ did not induce repression of root length (Fig. 2b). Phenotypic analysis of roots revealed that Co_3_O_4_-NPs (0.25, 0.5 and 1.0 mg/ml) treated groups exhibited a dose-dependent reduction in root length along with thickening, stunted growth and absence of root hairs. However, no such changes have been observed in bulk Co_3_O_4_ treated groups (Fig. 2c). Due to the non-significant effects of bulk Co_3_O_4_ on eggplant root length, we have excluded bulk Co_3_O_4_ in further experiments. Comparison of data with our recently published studies on NiO-NPs induced toxicity in tomato, suggested that Co_3_O_4_-NPs are more growth inhibitory at the highest concentration of 1.0 mg/ml and exhibited 2.14-fold greater repression of root length in eggplant. Thickening and stunted roots were observed at all treatment concentrations of Co_3_O_4_-NPs after 7 days, while the NiO-NPs treated tomato roots were found thickened and stunted only at greater concentrations of 1.5 and 2 mg/ml after 10 days of exposure [11], thus, suggesting the higher toxicity of Co_3_O_4_-NPs. The differences in toxicity of Co_3_O_4_-NPs over NiO-NPs can be attributed to the difference in seed size of the two plants. Indeed, the interactions of similar-sized NPs might be greater for the smaller seeds of eggplant as compared to the larger sized tomato seeds, largely due to greater surface-to-volume ratio of the small-sized seeds to that of the large-sized seeds [12]. These observations corroborate well with earlier literature suggesting greater phytotoxicity of rare earth oxide NPs to the smaller seeds (rape, cabbage, radish), as compared to the larger seeds of wheat [13]. Nonetheless, our results are in agreement with some recent findings, which have also demonstrated the repression of root length by CuO and Ag-NPs in *Arabidopsis thaliana* [3, 14]. Seed coat provides first line of defense to the seed from NPs during the germination. However, in this study we have demonstrated that seedling roots after piercing the seed coat, become the main organ to confront Co_3_O_4_-NPs. Therefore, the toxic symptoms were more evident in roots than in other parts of seedlings. In addition, seeds rough surface, electrostatic attraction and hydrophobic interactions contributed towards NPs adsorption on the seeds. Such adsorption may facilitate the release of ions from NPs and enhance the phytotoxicity [15].

### Co_3_O_4_-NPs uptake and translocation

**Fig. 3:**
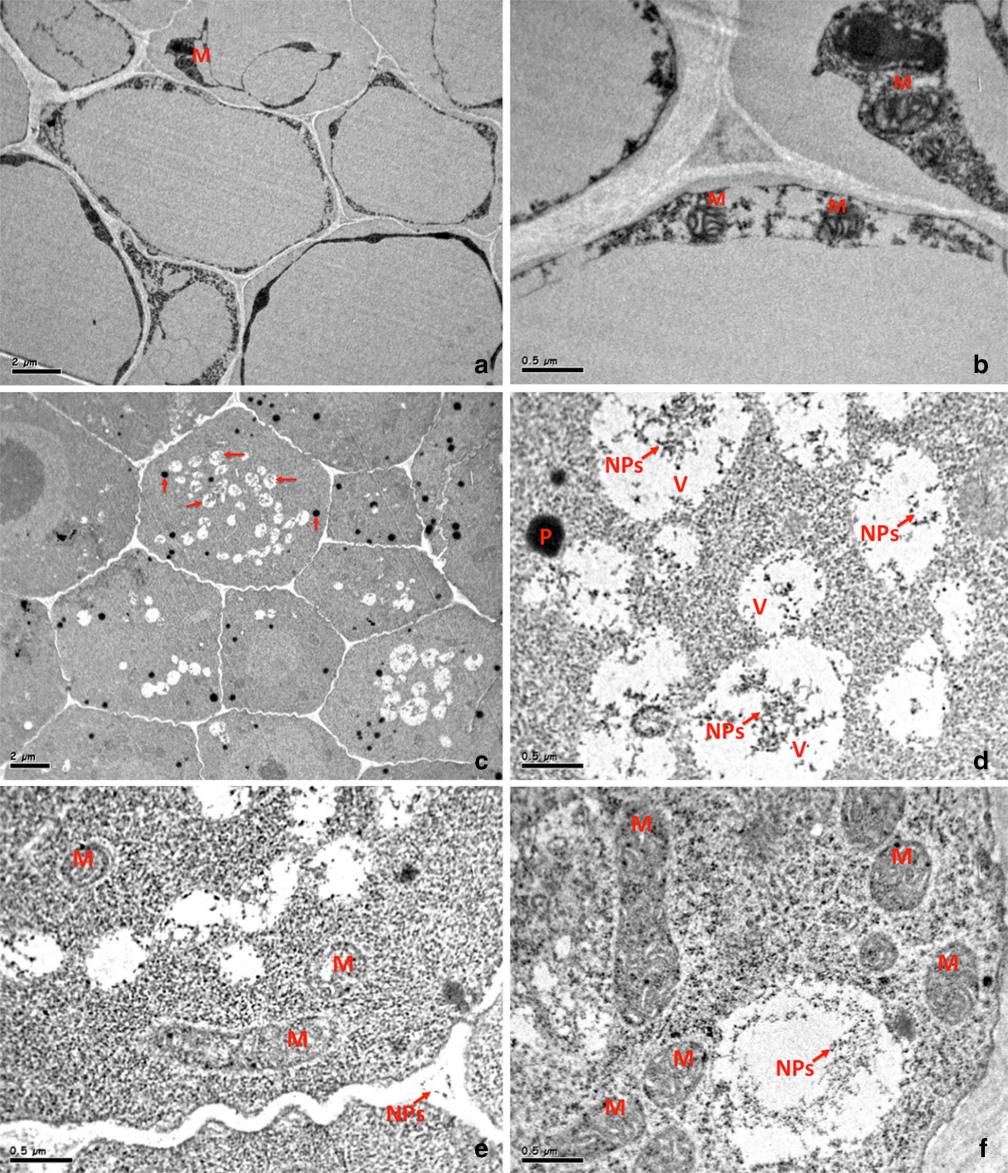
TEM images of control roots showing the triangular shaped parenchymal cells and mitochondria with integrated cristae, and no appearance of peroxisomes and vacuoles (**a**, **b**). Ultrastructural images of root sections from 1 mg/ml Co_3_O_4_-NPs treatment groups showing extensive vacuoles with NPs aggregates, mitochondria with degenerated cristae and abundance of peroxisomes (**c**, **d**, **f**). The extracellular region of parenchymatic cells showing the nanoparticles aggregates (**e**). Vacuoles (V), mitochondria (M), peroxisomes (P), nanoparticles (NPs)

To confirm the translocation of Co_3_O_4_-NPs, ultrathin sections of root tissues from elongation zone were evaluated by TEM (Fig. 3a–f). Root elongation zone consists of newly formed differentiated cells, which elongate at a rapid rate and exhibit enhanced cell cycle activity. Co_3_O_4_-NPs were observed in the cytoplasm and exterior region of confluent parenchymal cells (Fig. 3c, d). NPs were recognized as dark dots and aggregates entrapped in cell vacuoles. Our TEM data on Co_3_O_4_-NPs corroborate with recent studies exhibiting the translocation of Ag and ZnO-NPs *Triticum aestivum* and *Schoenoplectus tabernaemontani* [16, 17]. In addition, enhanced number of peroxisomes and mitochondria with degenerated cristae has also been encountered (Fig. 3c, e, f). These subcellular anomalies are found identical as observed in plants exposed to ozone [18]. Interestingly, the eukaryotic microorganism (yeast) has also demonstrated these common properties of peroxisome biogenesis as a consequence of mitochondrial dysfunction [19].

### Intracellular ROS production and mitochondrial dysfunction

**Fig. 4:**
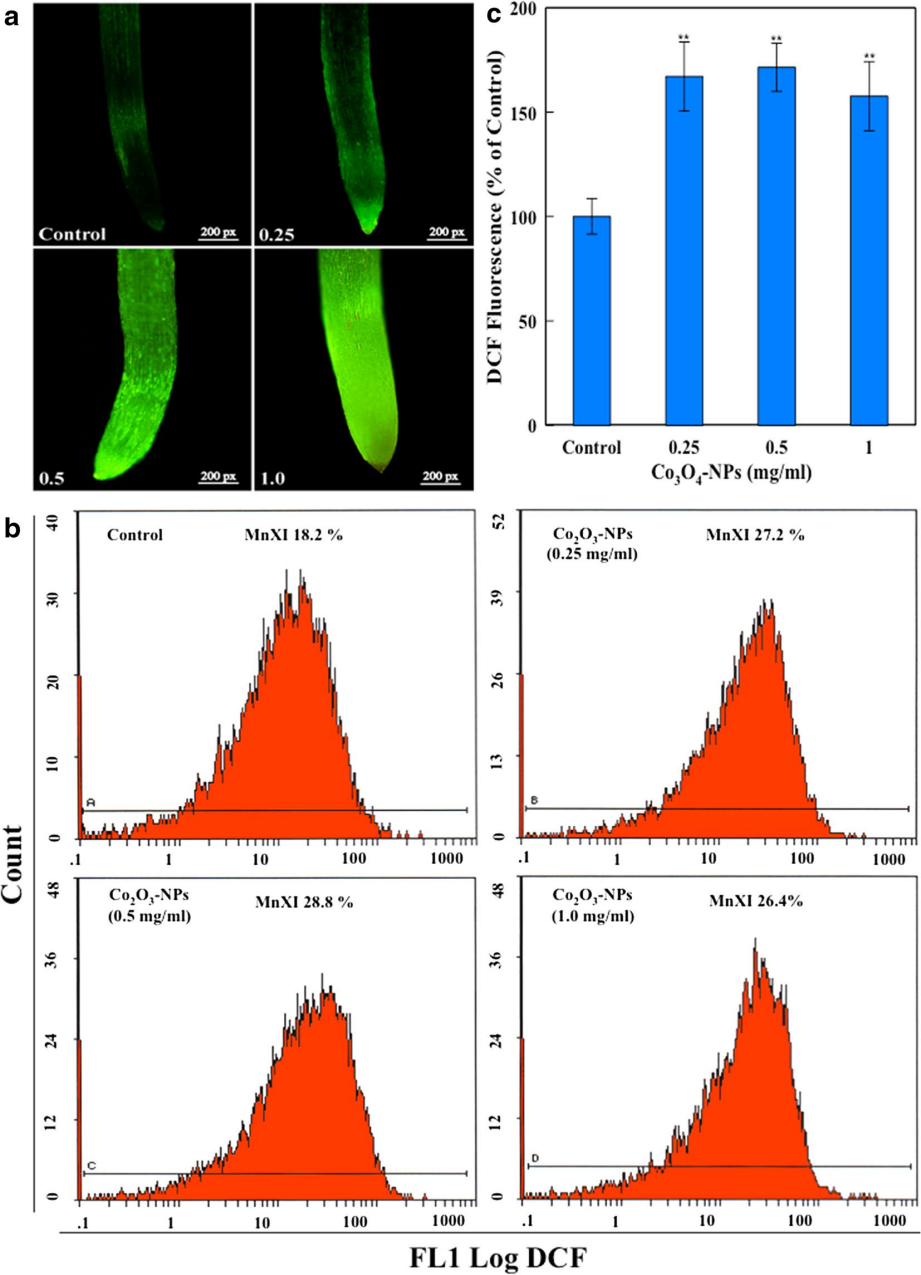
**a** Depicts DCF fluorescence in eggplant seedling roots with localization of ROS in root tip, area of elongation and differentiation. Representative flow cytometric images (**b**) and average DCF fluorescence (**c**) reaffirming intracellular ROS generation in protoplasts of Co_3_O_4_-NPs treated groups. MnXI = mean fluorescence intensity of DCF. (**p < 0.01 vs control)

Qualitative evaluation of Co_3_O_4_-NPs treated roots exhibited a conspicuous increase in DCF fluorescence. Eggplant roots stained with fluorescent dye DCFH-DA revealed a typical pattern of ROS localization as a function of Co_3_O_4_-NPs concentration. Compared to the untreated control, Co_3_O_4_-NPs at 0.25 and 0.5 mg/ml showed ROS localization around apical root meristem. Subsequently, the ROS localization becomes more intense towards root elongation zone at 1.0 mg/ml. (Figure 4a). Protoplasts quantitatively analyzed on flow cytometry also revealed higher levels of intracellular ROS. Relative to 100 % DCF fluorescence in control, Co_3_O_4_-NPs (0.25, 0.5 and 1.0 mg/ml) treatments resulted in 167.0, 177.4 and 157.6 % greater ROS generation (Fig. 4b, c). Our ROS data is in line with a previous study demonstrated oxidative stress in *Oryza sativa* treated with multiwalled carbon nanotubes [20]. It is well established that ROS rapidly interacts with distant cellular organelles and may result in DNA strand breaks, purine oxidation, protein modifications, protein-DNA cross links and mitochondrial damage [21, 22].

**Fig. 5:**
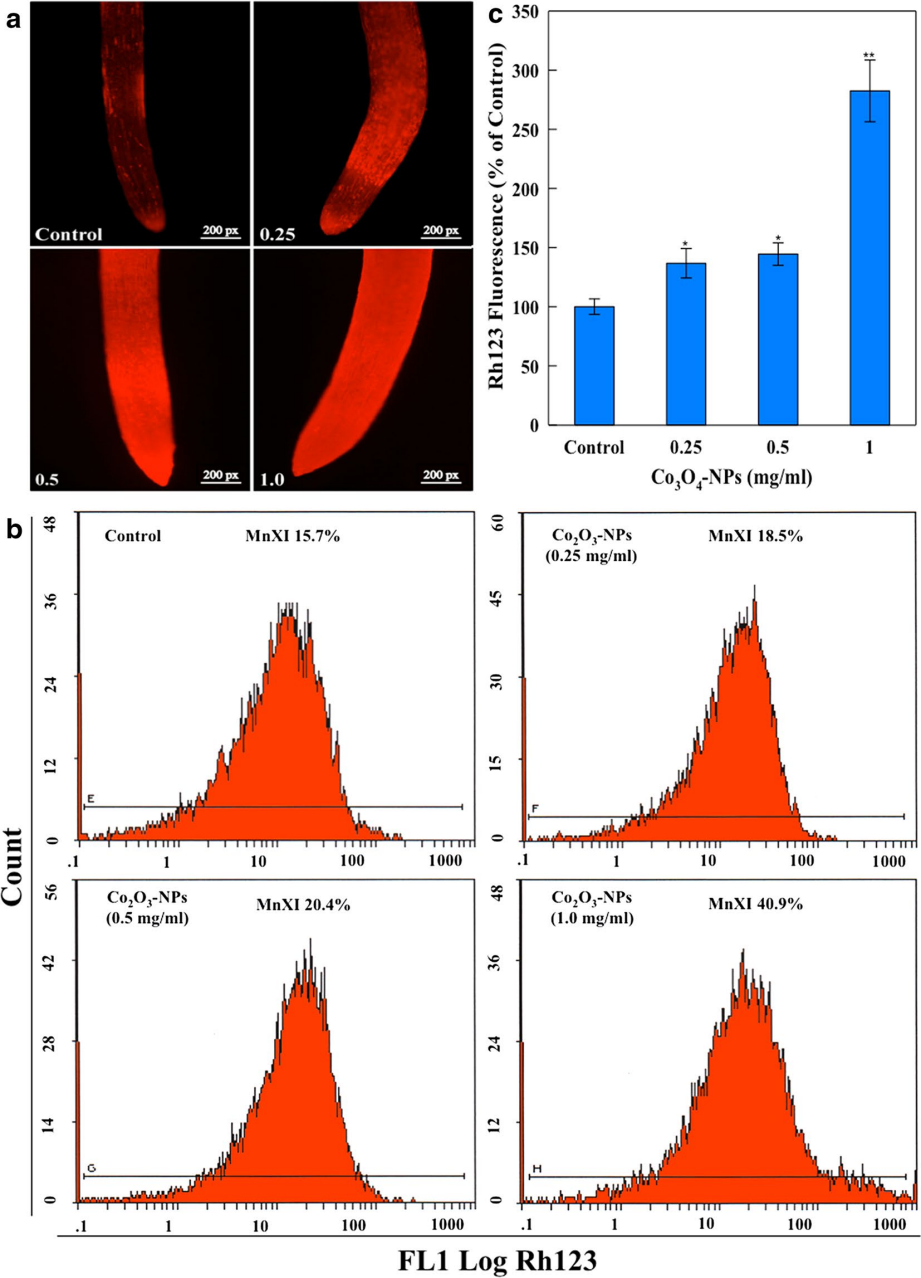
Qualitative analysis of Rh123 stained eggplant seedling roots showing the fluorescence enhancement upon Co_3_O_4_-NPs exposure while control shows normal bright fluorescence of Rh123 with no diffusion of dye throughout root length (**a**). The representative flow cytometric images (**b**) and average Rh123 fluorescence (**c**) reaffirming mitochondrial dysfunction in protoplasts of Co_3_O_4_-NPs treated groups. MnXI is the fluorescence intensity of Rh123 (*p < 0.05, **p < 0.01 vs control)

The interplay of ROS and mitochondria has further lead us to analyze Δ*Ψm* using mitochondrial specific dye Rh123. Qualitative evaluation of seedling roots from Co_3_O_4_-NPs treatment groups exhibited enhancement in the fluorescence intensity of Rh123 (Fig. 5a). A similar pattern of fluorescence enhancement has been observed in the protoplasts of Co_3_O_4_-NPs exposed groups. Flow cytometric analysis of protoplasts revealed 136.6, 144.2 and 282.4 % greater fluorescence at 0.25, 0.5 and 1.0 mg/ml concentrations (Fig. 5b, c). Mitochondrial dysfunction as a consequence of oxidative stress is an important factor in cell death [23, 24]. Fluorescence enhancement of Rh123 dye is related to the variation in Δ*Ψm*, which results as a consequence of swelling and shrinking properties of mitochondria [25]. Due to these morphological alterations, preferably by swelling, Rh123 leaks out from mitochondria to the cytoplasm [26]. Therefore, Co_3_O_4_-NPs mediated enhancement of Rh123 florescence confirm mitochondrial swelling in eggplant and the leakage of dye thus causing Rh123 hyperpolarization in cytoplasm.

### NO and esterase analysis

**Fig. 6:**
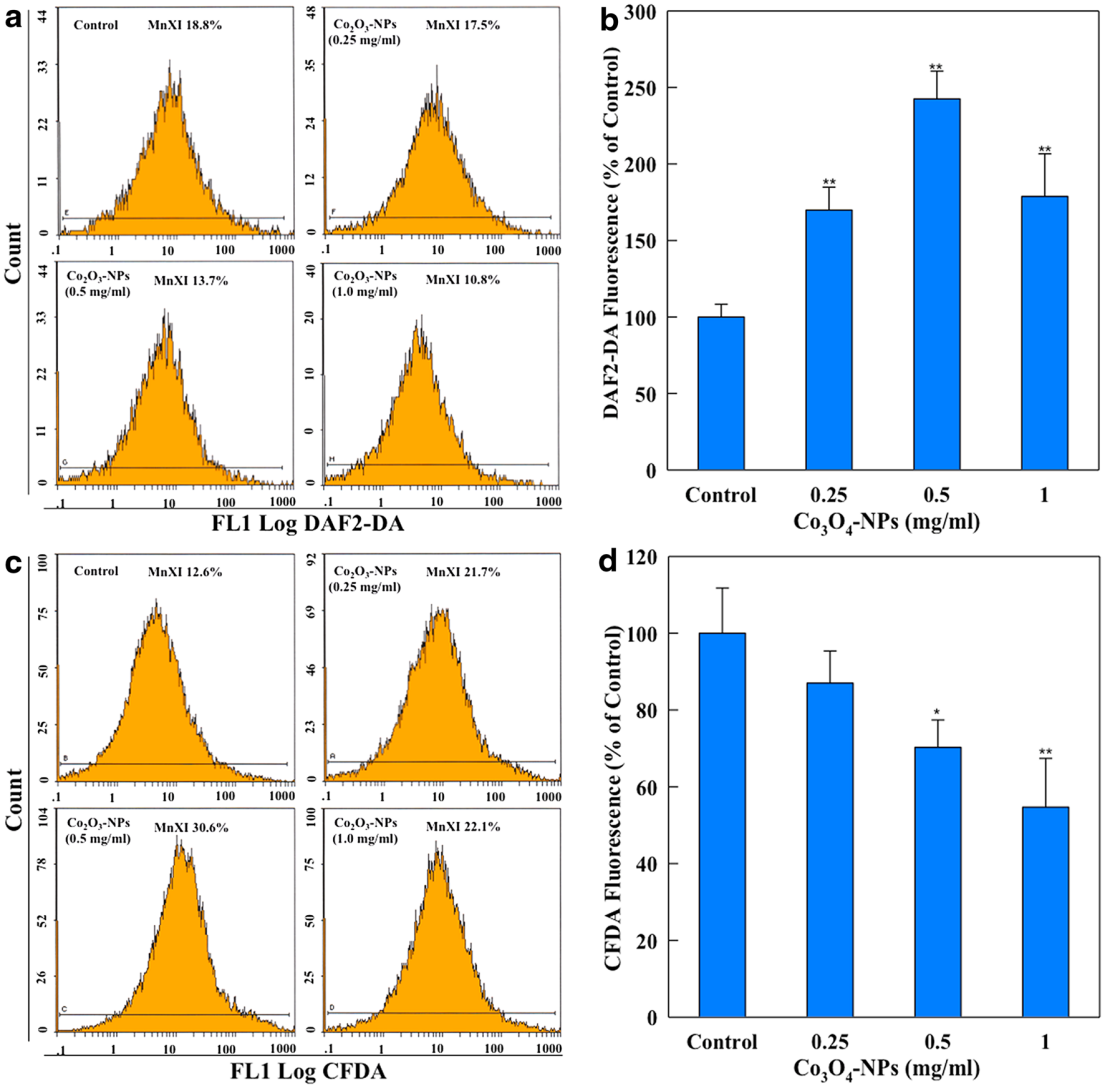
Representative flow cytometric images showing concentration dependent change in the mean fluorescence intensity (MnXI) of fluorescence probes DAF2-DA and CFDA specific for intracellular nitric oxide (NO) (**a**) and esterase activity (**c**) in eggplant protoplasts. Histograms shown in *panel* **b** and **d** represents changes in the mean ± SD of fluorescence of DAF2-DA and CFDA (MnXI) obtained from 10,000 protoplasts in three independent experiments (*p < 0.05, **p < 0.01 vs. control)

Flow cytometric analysis of Co_3_O_4_-NPs treated protoplasts exhibited an increase in intracellular NO generation. Relative to 100 % fluorescence in control, protoplasts isolated from 0.25, 0.5 and 1.0 mg/ml Co_3_O_4_-NPs treated cells showed 169.8, 242.5 and 178.7 % (p < 0.01) higher fluorescence of DAF2-DA (Fig. 6a, b). Our data is in line with earlier studies, which also suggested an elevated level of NO generation in *Lupinus luteus* and *Glycine max* exposed to Cd^++^ [27, 28]. NO has been regarded as an effective inducer of cell death by interfering with mitochondria functionality and promoting imbalance between ROS generation and scavenging [28]. Furthermore, NO has been described as a small signaling molecule in plants taking part in several events throughout the life cycle, as well as in defense against biotic and abiotic stresses [29].

We further investigated the level of intracellular esterases, regarded as a prevalent biomarker to assess the viability of cells [30]. Relative to 100 % fluorescence of CFDA in control, Co_3_O_4_-NPs treated root cells at the concentrations of 0.5 and 1.0 mg/ml exhibited a decline by 29.7 and 45.2 %, respectively. However, no significant change in the esterase level has been observed at 0.25 mg/ml (Fig. 6c, d). CFDA fluoresces strongly when de-esterified to carboxyfluorescein (CF). Conversion to CF by cells indicates the integrity of the plasma membrane. An intact membrane prevents leakage of the polar dye into the medium and maintains cytoplasmic milieu, which is needed to support esterase activity [31]. Therefore, the suppressed esterase level in this study primarily suggests that Co_3_O_4_-NPs induce membrane damage in protoplasts and in mitochondria of eggplant cells.

### Co_3_O_4_-NPs induced DNA damage

**Fig. 7:**
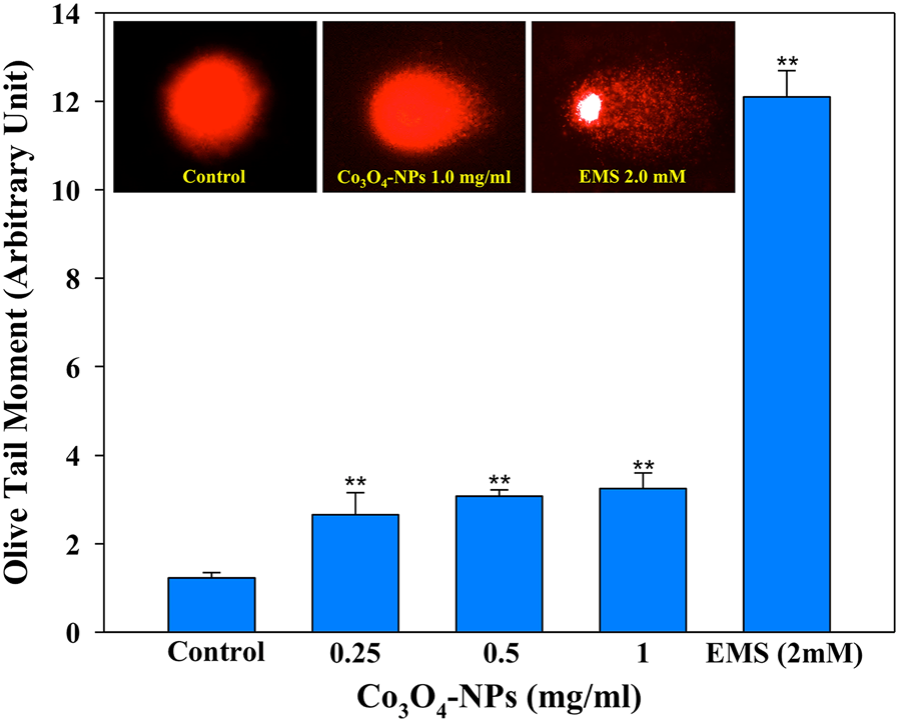
*Inset* shows the representative epi-fluorescence comet images of Co_3_O_4_-NPs-induced DNA damage in eggplant nuclei analyzed by alkaline single cell gel electrophoresis. Quantitative analysis of DNA damage using Comet assay IV software shows a concentration dependent single strand breaks in eggplant nuclei. Each *data point* represents ± SD value of three independent experiments done in duplicate comet slides. EMS; Ethyl methanesulfonate (2 mM) taken as positive control

Alkaline single cell gel electrophoresis (comet assay) data showed Co_3_O_4_-NPs-induced single strand breaks in DNA of eggplant cells. The representative digitized image of comet tail in Co_3_O_4_-NPs (1.0 mg/ml) treated group clearly demonstrates the extent of broken DNA liberated from head of the comet during electrophoresis (Fig. 7 inset). However, under identical condition the control cell exhibited round boundary of nuclear head DNA. A conspicuous tail in positive control ethyl methanesulfonate (EMS 2 mM) confirms the proper functioning of comet setup (Fig. 7 inset). Compared to the olive tail moment (OTM) value of 1.22 ± 0.12 in control, the quantitative data obtained from 150 cells of Co_3_O_4_-NPs (0.25, 0.5 and 1.0 mg/ml) treatment groups exhibited an increase of 2.6 ± 0.49, 3.0 ± 0.14 and 3.2 ± 0.35 (p < 0.01), respectively, over control (Fig. 7). The DNA damaging activity of Co_3_O_4_-NPs has been found to be similar to the DNA damaging effect of TiO_2_ and ZnO-NPs reported in *A. cepa* [32]. Thus, the results substantiate the hypothesis that plant-NPs interaction is associated with the genotoxicity and concurs well with the earlier reports. Metal oxides as well as metals are involved in ROS formation, which can interact directly or indirectly with plant DNA to induce strand breaks [33, 34]. However, on the basis of our data we suggest that Co_3_O_4_-NPs may have indirectly induce DNA damage in eggplant, as evident from increase in the ROS, mitochondrial membrane damage and elevated esterase level. On the contrary, the NiO-NPs may have directly interacted with the DNA to induce non-repairable heavy damage; as a result an increasing number of apoptotic/necrotic cells were appeared in the comet assay of tomato cells [11]. Thus, an in-depth investigation is warranted in order to ascertain the extent and nature of direct interactions of Co_3_O_4_-NPs using spectrofluorometric, spectrophotometric biophysical and bioinformatics tools.

### Flow cytometric analysis of apoptosis

**Fig. 8:**
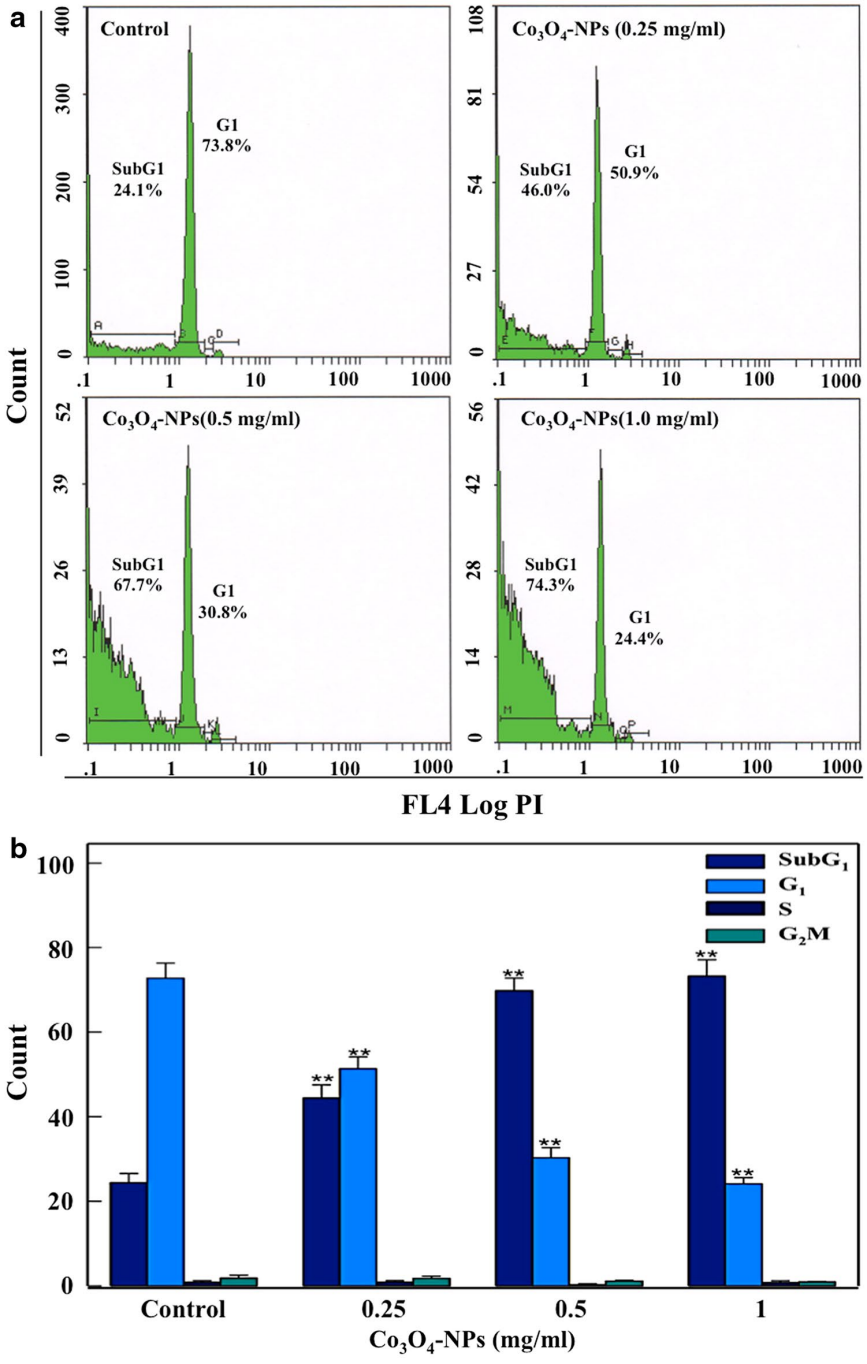
Effect of Co_3_O_4_-NPs on cell cycle of eggplant. **a** Flow images exhibiting disruption of cell cycle peaks with increasing concentrations of Co_3_O_4_-NPs. **b** Individual histograms represents the mean ± SD values of different phases of cell cycle

In order to assess Co_3_O_4_-NPs induced cell death, the apoptotic events occurred in treated eggplants cells were captured by use of a flow cytometer. The propiodium iodide (PI) stained nuclei of Co_3_O_4_-NPs treatment groups indicated a concentration dependent increase in apoptotic sub-G1 peak (Fig. 8a). In comparison with 24.4 % of background apoptotic cells in control, Co_3_O_4_-NPs at 0.25, 0.5 and 1.0 mg/ml exhibited 44.4, 69.8 and 73.2 % of apoptotic population (Fig. 8b). A similar mode of cell death has also been observed in our earlier report on tomato cells exposed to NiO-NPs [11]. Furthermore, our ongoing studies on cultured human cell lines with Co_3_O_4_-NPs have demonstrated that these NPs have the potential to induce apoptosis in the human cell lines (data unpublished). It is widely accepted that apoptotic mechanisms in plants and animals share some common components leading to conserved cellular events [35, 36]. Therefore, it is likely that eggplant root cells may activate a similar mechanism of cell death.

## Conclusions

In conclusion, our study demonstrates that eggplant exposed to Co_3_O_4_-NPs exhibited a significant repression of root growth due to phytotoxic properties of NPs. Ultrastructural analysis suggests the subcellular localization of Co_3_O_4_-NPs to induce organelles damage. Fluorescence imaging and flow cytometric data supported the fact that ROS plays a crucial role in mitochondrial damage to trigger apoptosis in eggplant. Higher level of NO and mitochondrial membrane damage revealed that Co_3_O_4_-NPs trigger cell death in eggplant via mitochondrial swelling and stimulation of NO signaling pathway. Furthermore, the depletion of esterase activities in cells could serve as a useful biomarker of Co_3_O_4_-NPs mediated cellular stress. Presumably, the root cells exhibiting NPs induced cell death may share some similar apoptotic characters, as observed in animals. Nonetheless, a deep investigation is warranted on transcriptome analysis to investigate the possible connection between different apoptotic factors. Finally, we conclude with the remark that the current findings will provide strong background to explore NPs induced toxicity at field or farm level to determine a realistic exposure scenario for other crops.

## Methods

### Characterization of nanoparticles

Co_3_O_4_-NPs (1 mg/ml) (Cat. No. 637025, Sigma-Aldrich, St. Louis, MO, USA) were sonicated in ultrapure water for 10 min at 50 W, and the solution was dropped on copper grids of the transmission electron microscope (TEM). A total of six grids of Co_3_O_4_-NPs were prepared and subjected to TEM analysis at 200 keV. Characterizations of Co_3_O_4_-NPs were further done by analyzing the surface topography of powdered Co_3_O_4_-NPs using atomic force microscope (AFM) (Veeco Instruments, USA) in non-contact tapping mode. The topographical images were obtained in tapping mode with a resonance frequency of 218 kHz. Characterization of Co_3_O_4_-NPs was further done in liquid environment by measuring the dynamic light scattering (DLS) and zeta (ζ)-potential using Zetasizer 2000 (ZetaSizer-HT, Malvern, UK). Briefly, Co_3_O_4_-NPs stock suspension of 10 µg/ml was prepared in ultrapure water, sonicated for 15 min at 40 W and the solutions were analyzed for DLS and ζ-potential, values presented were the average of 10 readings.

### Eggplant root length retardation by Co_3_O_4_-NPs and bulk Co_3_O_4_

To determine the phytotoxicity in *Solanum melongena* L. cv. Violetta lunga 2. (eggplant), we have selected Co_3_O_4_-NPs and its bulk counterpart Co_3_O_4_ (Cat. No. 221643, Sigma-Aldrich, St. Louis, MO, USA). Seeds of eggplant were surface sterilized in Clorox solution (5 % v/v) for 10 min followed by through washing with distilled water. The exposure concentrations of Co_3_O_4_-NPs were selected from initial experiments based on the root elongation assay. For each set of experiment, 20 seeds were treated with 0.025, 0.05, 0.1, 0.25, 0.5 and 1.0 mg/ml of Co_3_O_4_-NPs (<50 nm) and bulk Co_3_O_4_ (<10 µm) suspensions for 2 h on a rotary shaker. Parallel untreated controls were run under identical conditions. After treatment, seeds were thoroughly washed with distilled water and transferred to Petri dishes containing wet filter papers. Petri dishes were kept in the growth chamber at 25 ± 2 °C for 7 days for seed germination and growth.

### Uptake of Co_3_O_4_-NPs

Subcellular changes in eggplant root cells were analyzed by use of TEM. Root tissues from control and Co_3_O_4_-NPs (1.0 mg/ml) groups were fixed in glutaraldehyde for 10 min; followed by re-suspension of root sections in OsO_4_ (1 %) for 1 h at 4 °C. An additional incubation of 1 h was given for each section in 2 % aqueous uranyl acetate pursued by the dehydration of sections using ascending grade of ethanol. Root sections were finally embedded in low viscosity araldite resin and ultrathin sections of 80 nm were made from elongation zone for TEM analysis under high vacuum (100 kV).

### Flow cytometric analysis of intracellular ROS and mitochondrial dysfunction (Δ*Ψm*)

For qualitative analysis of ROS and Δ*Ψm*, Co_3_O_4_-NPs treated seedling roots were stained with 2′,7′-dichlorofluorescin diacetate (DCFH-DA) (0.25 µM) and 1 µg/ml of rhodamine 123 (Rh123) for 15 min. Roots were washed three times with PBS, and images were captured using a fluorescence microscope (Nikon Eclipse 80i, Japan) [10, 11]. Quantitative estimation of intracellular ROS and Δ*Ψm* was done in protoplasts isolated from control and Co_3_O_4_-NPs treated groups according to the method of Imanishi et al. [37], with slight modification [11]. In brief, 10 root tissues from each of control and treated samples were incubated in 1.5 % cellulose, 0.5 % pectinase (Sigma) in Galbraith buffer (45 mM MgCl_2_, 30 mM sodium citrate, 20 mM MOPS, 0.1 % (v/v) Triton X-100, pH 7.0) for overnight in the dark at 26 °C. The digested root/enzyme solution was filtered through a 100 µm sieve and viable cells recovered by flotation after centrifugation in Galbraith buffer. Centrifugation and recovery steps of intact cells were repeated thrice to remove enzymes. Protoplasts from control and Co_3_O_4_-NPs treated groups were separately stained with DCFH-DA (5 µM) and Rh123 (5 µg/ml) for 1 h in the dark at room temperature. Fluorescence of 10,000 protoplasts from each dye treatment was recorded on Beckman Coulter flow cytometer (Coulter Epics XL/Xl-MCL, USA) following our previously described methods [38, 39].

### Assessment of NO and esterase activity by flow cytometer

Intracellular NO and esterase activities in protoplasts were measured using flow cytometry. Protoplasts isolated from Co_3_O_4_-NPs treated, and untreated eggplant seedling roots were washed twice with PBS and incubated with NO specific dye 4,5-diaminofluorescein diacetate (DAF2-DA, 5 µM) and esterase specific carboxyfluorescein diacetate (CFDA, 5 µM) for 60 min in the dark at room temperature. The protoplast suspensions were pelleted, followed by two successive washes with PBS at 3000 rpm at 4 °C for 4 min. The protoplasts were re-suspended in a final volume of 500 µl of PBS and the fluorescence of DAF2-DA and CFDA was measured in 10,000 protoplasts using a flow cytometer at log scale (FL-1, 530 nm).

### DNA damage analysis by alkaline comet assay

The comet assay was performed to analyze the DNA damage in nuclei following the method described by Faisal et al. [10]. Nuclei were isolated by chopping the root tissues using a sharp scalpel blade in 1.0 ml of Galbraith buffer (45 mM MgCl_2_, 30 mM sodium citrate, 20 mM MOPS, 0.1 % (v/v) Triton X-100, pH 7.0). Comet slides were prepared following our previously described method [40].

### Flow cytometric analysis of apoptosis in eggplant

Apoptosis analysis in eggplant roots was done using flow cytometry following our previously described method [11]. Nuclei suspensions (1.0 ml) from control and Co_3_O_4_-NPs treated groups were stained with 10 µg/ml of DNA intercalating fluorescent dye (propidium iodide, PI) and RNAase A (50 µg/ml) solutions for 10 min on ice. Red fluorescence of 100,000 events of PI stained nuclei were acquired in FL4 Log channel through a 675 nm band-pass filter [38]. Data were analyzed excluding the cell debris, characterized by a low FSC/SSC, using Beckman Coulter flow cytometer (Coulter Epics XL/Xl-MCL, USA and System II Software, Version 3.0).

### Statistical analysis

Data were expressed as mean ± standard deviation (SD) for the values obtained from at least three independent experiments using 20 seeds/concentration. Statistical analysis was performed by one-way analysis of variance (ANOVA) followed by Dunnett’s multiple comparisons test (Sigma Plot 11.0, USA). The level of statistical significance chosen was p < 0.05, unless otherwise stated.
